# Using Nomograms to Predict the PPCs of Patients With Diffuse Peritonitis Undergoing Emergency Gastrointestinal Surgery

**DOI:** 10.3389/fmed.2021.705713

**Published:** 2021-12-23

**Authors:** Qiong Xue, Yu Zhu, Ying Wang, Jian-Jun Yang, Cheng-Mao Zhou

**Affiliations:** ^1^Department of Anesthesiology, Pain and Perioperative Medicine, First Affiliated Hospital of Zhengzhou University, Zhengzhou, China; ^2^Department of Scientific Research, Zhaoqing Medical College, Zhaoqing, China

**Keywords:** nomogram, PPC, diffuse peritonitis, emergency, gastrointestinal surgery

## Abstract

**Objective:** To develop and validate a nomogram model for predicting postoperative pulmonary complications (PPCs) in patients with diffuse peritonitis undergoing emergency gastrointestinal surgery.

**Methods:** We used the least absolute shrinkage and selection operator (LASSO) regression model to analyze the independent risk factors for PPCs in patients with diffuse peritonitis who underwent emergency gastrointestinal surgery. Using R, we developed and validated a nomogram model for predicting PPCs in patients with diffuse peritonitis undergoing emergency gastrointestinal surgery.

**Results:** The LASSO regression analysis showed that AGE, American Society of Anesthesiologists physical status classification (ASA), DIAGNOSIS, platelets (on the 3rd day after surgery), cholesterol (on the 3rd day after surgery), ALBUMIN (on the first day after surgery), and preoperative ALBUMIN were independent risk factors for PPCs in patients with diffuse peritonitis undergoing emergency gastrointestinal surgery. The area under the curve (AUC) value of the nomogram model in the training group was 0.8240; its accuracy was 0.7000, and its sensitivity was 0.8658. This demonstrates that the nomogram has a high prediction value. Also in the test group, the AUC value of the model established by the variables AGE, ASA, and platelets (on the 3rd day after surgery), cholesterol (on the 3rd day after surgery), ALBUMIN (on the first day after surgery), and preoperative ALBUMIN was 0.8240; its accuracy was 0.8000; and its specificity was 0.8986. In the validation group, the same results were obtained. The results of the clinical decision curve show that the benefit rate was also high.

**Conclusion:** Based on the risk factors AGE, ASA, DIAGNOSIS, platelets (on the 3rd day after surgery), cholesterol (on the 3rd day after surgery), ALBUMIN (on the first day after surgery), and preoperative ALBUMIN, the nomogram model established in this study for predicting PPCs in patients with diffuse peritonitis undergoing emergency gastrointestinal surgery has high accuracy and discrimination.

## Introduction

Complicated intra-abdominal infections extend beyond organs and may result in localized or diffused peritonitis (1). Early prognostic assessment and testing for diffuse peritonitis are essential for evaluating disease severity and for providing optimal treatment (2). Postoperative pulmonary complications (PPCs), an important cause of morbidity after upper abdominal surgery, resulting in a significant increase in hospital stays and medical costs (3).

Postoperative pulmonary complications include conditions, such as atelectasis, pleural effusion, pulmonary infection, bronchospasm, acute respiratory failure, and acute respiratory distress syndrome, which are important causes of death and morbidity after major surgery (4).

Therefore, it is of great significance to the prevention and treatment of PPCs to explore risk factors for PPCs after upper abdominal surgery and to screen high-risk patients. These are both hot topics in clinical research. Thus, the individualized prediction of the risk of PPCs is the crux of PPC prevention and treatment. A nomogram model, which integrates relevant risk factors, is able to individually predict adverse clinical events. Therefore, by analyzing the clinical data of patients with diffuse peritonitis undergoing emergency gastrointestinal surgery, this study explores these patients' independent risk factors for PPCs. The aim was to develop and validate individualized prediction of PPCs in patients with diffuse peritonitis undergoing emergency gastrointestinal surgery, assist in the clinical identification of high-risk patients, and provide scientific guidance for the individualized prevention and treatment of PPCs.

## Materials and Methods

### Patients

We retrospectively analyzed the medical records of critically ill patients (≥18 years old) with diffuse peritonitis who had undergone emergency surgery. Patients with surgery for acute appendicitis, acute cholecystitis, or necrotizing pancreatitis were excluded; patients who had died within the first 7 days after surgery were excluded; and patients with unmeasured total cholesterol levels either before or 1, 3, and 7 days after surgery were excluded as well. Since it was a retrospective study, the requirement for informed consent was waived.

Patient data of the training group and the test group were obtained from BioStudies Database (https://www.ebi.ac.uk/biostudies/studies/S-EPMC6034864), while patient data of the validation group were obtained from the First Affiliated Hospital of Zhengzhou University.

### Perioperative Variables

The analysis included the following variables: age, sex, body mass index, American Society of Anesthesiologists physical status classification (ASA) score, lesion location, diagnosis, perioperative shock, preoperative laboratory findings, postoperative complications, and type of surgery. Pulmonary complications were defined as the presence of one or more of the following postoperative symptoms: pneumonia, atelectasis, pleural effusion, or acute respiratory distress syndrome.

### Statistical Analysis

We used R version 3.1.3 (http://www.R-project) and Empower Stats (http://www.empowerstats.com/cn/download.html) for our analysis. The R glmnet package was called for least absolute shrinkage and selection operator (LASSO) regression analysis, and lambda was screened through 10-fold cross-validations. The larger the lambda, the smaller the model. We developed the nomogram prognostic model based on independent prognostic factors and assessed the clinical net benefit by decision curve analysis (DCA). To create each nomogram, we used the RMS package in R. Each individual's total score was calculated according to the nomogram. Then, we performed a diagnostic test on the total score and outcome. We calculated the area under the receiver operating characteristic curve (ROC) curve and its 95% CI, setting a total score corresponding to the sum of sensitivity and specificity as the cut-off point.

## Results

### Analysis of Factors Related to the Prognosis of the Patients

A total of 1,126 patients were enrolled in this study. The mean age of the PPCs in the training group was 66.2 ± 13.6 years, and the mean age of the patients without PPCs was 58.0 ± 16.5 years. The characteristics of the remaining baseline are shown in Table 1.

**Table 1:** Characteristics of patients in the training, the test, and the validation groups.

	**Training**			**Test**			**Validation**		
**Pulmory**	**No**	**Yes**	***P*-value**	**No**	**Yes**	***P*-value**	**No**	**Yes**	***P*-value**
N	591	149		148	38		131	69	
age (year)	58.0 ± 16.5	66.2 ± 13.6	<0.001	59.3 ± 14.6	63.4 ± 17.5	0.050	58.4 ± 17.5	64.0 ± 15.9	0.032
HB.1, g/dL	12.2 ± 2.4	11.3 ± 2.4	<0.001	12.2 ± 2.3	11.0 ± 2.2	0.003	12.0 ± 2.2	11.4 ± 2.3	0.056
HB.0, g/dL	11.6 ± 2.1	10.9 ± 2.4	<0.001	11.6 ± 2.1	10.8 ± 2.2	0.043	11.7 ± 2.1	11.6 ± 2.3	0.871
HB+0.1, g/dL	10.7 ± 2.0	9.9 ± 1.8	<0.001	10.5 ± 2.0	10.0 ± 1.8	0.232	11.0 ± 2.0	10.8 ± 2.1	0.439
HB.3, g/dL	10.2 ± 1.6	9.7 ± 1.4	0.005	10.2 ± 1.7	9.7 ± 1.7	0.069	10.3 ± 1.9	10.1 ± 2.0	0.290
PLATELET.1,10^3^/μL	276.0 ± 129.0	268.4 ± 154.6	0.144	283.2 ± 129.3	256.4 ± 114.2	0.402	264.4 ± 100.8	202.8 ± 76.8	<0.001
PLATELET.0,10^3^/μL	244.9 ± 121.8	218.8 ± 136.4	0.002	256.4 ± 123.3	219.1 ± 102.7	0.074	237.2 ± 79.9	195.3 ± 82.8	0.006
PLATELET.1.1,10^3^/μL	224.8 ± 112.4	175.4 ± 110.1	<0.001	228.7 ± 104.0	180.3 ± 104.1	0.004	220.7 ± 98.7	161.3 ± 90.9	<0.001
PLATELET.3,10^3^/μL	223.8 ± 113.3	162.9 ± 111.9	<0.001	228.2 ± 100.4	146.3 ± 120.2	<0.001	210.4 ± 94.9	155.8 ± 87.9	<0.001
CHOLESTEROL.1, mg/dL	144.7 ± 50.5	120.2 ± 42.4	<0.001	139.0 ± 45.1	111.8 ± 49.1	<0.001	201.9 ± 471.1	151.7 ± 47.2	0.202
CHOLESTEROL.0, mg/dL	109.6 ± 49.3	79.0 ± 35.6	<0.001	107.2 ± 40.9	73.6 ± 45.1	<0.001	113.9 ± 27.4	113.3 ± 13.1	0.976
CHOLESTEROL.1.1, mg/dL	89.9 ± 39.1	63.6 ± 24.4	<0.001	88.0 ± 35.0	56.9 ± 27.4	<0.001	89.5 ± 17.9	87.6 ± 7.1	0.138
CHOLETEROL.3, mg/dL	101.7 ± 37.2	74.3 ± 26.4	<0.001	100.5 ± 35.9	69.1 ± 30.9	<0.001	118.5 ± 26.3	116.7 ± 12.4	0.529
ALBUMIN.1, g/dL	3.5 ± 0.7	3.0 ± 0.6	<0.001	3.5 ± 0.6	2.9 ± 0.6	<0.001	3.3 ± 0.6	3.0 ± 0.5	0.019
ALBUMIN.0, g/dL	2.8 ± 0.7	2.3 ± 0.6	<0.001	2.7 ± 0.7	2.3 ± 0.7	<0.001	3.0 ± 0.7	2.9 ± 0.5	0.192
ALBUMIN.1.1, g/dL	2.8 ± 0.4	2.7 ± 0.4	0.013	2.8 ± 0.4	2.8 ± 0.4	0.341	2.6 ± 0.5	2.4 ± 0.5	0.012
ALBUMIN.3, g/dL	2.9 ± 0.4	2.8 ± 0.3	<0.001	3.0 ± 0.3	2.8 ± 0.3	0.002	2.9 ± 0.4	2.8 ± 0.4	0.026
T-BILIRUBIN.1, mg/dL	1.0 ± 1.3	1.2 ± 1.3	0.071	0.8 ± 0.6	1.0 ± 0.7	0.078	1.3 ± 1.4	1.7 ± 1.7	0.061
T-BILIRUBIN.0, mg/dL	1.0 ± 0.9	1.3 ± 1.3	0.312	0.9 ± 0.6	1.2 ± 1.2	0.720	1.1 ± 0.7	1.3 ± 1.3	0.206
T-BILIRUBIN.1.1, mg/dL	1.1 ± 1.0	1.5 ± 1.6	0.043	0.9 ± 0.6	1.7 ± 1.6	0.015	1.2 ± 1.2	1.5 ± 1.6	0.069
T-BILIRUBIN.3, mg/dL	1.0 ± 1.2	1.7 ± 2.0	<0.001	0.9 ± 0.8	2.0 ± 1.8	<0.001	1.2 ± 1.0	1.7 ± 1.6	0.010
sex			0.276			0.263			0.500
Male	356 (60.2%)	97 (65.1%)		99 (66.9%)	29 (76.3%)		81 (61.8%)	46 (66.7%)	
Female	235 (39.8%)	52 (34.9%)		49 (33.1%)	9 (23.7%)		50 (38.2%)	23 (33.3%)	
ASA			<0.001			0.123			<0.001
1	202 (34.2%)	51 (34.2%)		64 (43.2%)	14 (36.8%)		32 (24.4%)	4 (5.8%)	
2	199 (33.7%)	27 (18.1%)		46 (31.1%)	8 (21.1%)		55 (42.0%)	23 (33.3%)	
3	156 (26.4%)	43 (28.9%)		33 (22.3%)	13 (34.2%)		43 (32.8%)	37 (53.6%)	
4	33 (5.6%)	27 (18.1%)		5 (3.4%)	2 (5.3%)		1 (0.8%)	4 (5.8%)	
5	1 (0.2%)	1 (0.7%)		0 (0.0%)	1 (2.6%)		0 (0.0%)	1 (1.4%)	
HTN			0.014			0.797			0.003
No	408 (69.0%)	87 (58.4%)		98 (66.2%)	26 (68.4%)		99 (75.6%)	38 (55.1%)	
Yes	183 (31.0%)	62 (41.6%)		50 (33.8%)	12 (31.6%)		32 (24.4%)	31 (44.9%)	
DM			0.002			0.416			0.369
No	513 (86.8%)	114 (76.5%)		135 (91.2%)	33 (86.8%)		116 (88.5%)	58 (84.1%)	
Yes	78 (13.2%)	35 (23.5%)		13 (8.8%)	5 (13.2%)		15 (11.5%)	11 (15.9%)	
CRF			0.241			0.966			1.000
No	565 (95.6%)	139 (93.3%)		136 (91.9%)	35 (92.1%)		130 (99.2%)	68 (98.6%)	
Yes	26 (4.4%)	10 (6.7%)		12 (8.1%)	3 (7.9%)		1 (0.8%)	1 (1.4%)	
PUL.TBC			0.583			0.392			1.000
No	551 (93.2%)	137 (91.9%)		142 (95.9%)	35 (92.1%)		130 (99.2%)	69 (100.0%)	
Yes	40 (6.8%)	12 (8.1%)		6 (4.1%)	3 (7.9%)		1 (0.8%)	0 (0.0%)	
Maligncy			0.054			0.381			<0.001
No	290 (49.1%)	60 (40.3%)		70 (47.3%)	21 (55.3%)		103 (78.6%)	37 (53.6%)	
Yes	301 (50.9%)	89 (59.7%)		78 (52.7%)	17 (44.7%)		28 (21.4%)	32 (46.4%)	
Diagnosis			<0.001			0.873			0.345
Perforation	417 (70.6%)	116 (77.9%)		108 (73.0%)	27 (71.1%)		70 (53.4%)	34 (49.3%)	
Strangulation	134 (22.7%)	14 (9.4%)		22 (14.9%)	6 (15.8%)		53 (40.5%)	26 (37.7%)	
Anastomotic leakage	15 (2.5%)	8 (5.4%)		7 (4.7%)	1 (2.6%)		3 (2.3%)	2 (2.9%)	
Acute mesenteric ischemia	25 (4.2%)	11 (7.4%)		11 (7.4%)	4 (10.5%)		5 (3.8%)	7 (10.1%)	
Location.of.lesion			0.908			0.578			0.342
Stomach	89 (15.1%)	24 (16.1%)		25 (16.9%)	3 (7.9%)		35 (26.7%)	20 (29.0%)	
Duodenum	40 (6.8%)	12 (8.1%)		7 (4.7%)	2 (5.3%)		11 (8.4%)	3 (4.3%)	
Jejunum and ileum	204 (34.5%)	48 (32.2%)		50 (33.8%)	15 (39.5%)		54 (41.2%)	35 (50.7%)	
Colon and rectum	258 (43.7%)	65 (43.6%)		66 (44.6%)	18 (47.4%)		31 (23.7%)	11 (15.9%)	
Surgery.type			0.051			0.141			0.015
Primary repair	149 (25.2%)	37 (24.8%)		42 (28.4%)	8 (21.1%)		46 (35.1%)	24 (34.8%)	
Small bowel resection with anastomosis	30 (5.1%)	10 (6.7%)		10 (6.8%)	0 (0.0%)		28 (21.4%)	7 (10.1%)	
Ileo- or jejunostomy	145 (24.5%)	28 (18.8%)		29 (19.6%)	11 (28.9%)		31 (23.7%)	30 (43.5%)	
Hartmann‘s procedures or colostomy	94 (15.9%)	14 (9.4%)		24 (16.2%)	3 (7.9%)		14 (10.7%)	6 (8.7%)	
Colon resection with anastomosis	88 (14.9%)	27 (18.1%)		24 (16.2%)	7 (18.4%)		10 (7.6%)	1 (1.4%)	
Gastrectomy (subtotal or total)	85 (14.4%)	33 (22.1%)		19 (12.8%)	9 (23.7%)		2(1.5%)	1(1.4%)	

*Results in the table: Mean + SD/N(%). Hb, hemoglobin; HTN, hypertension; DM, diabetes mellitus; CRF, chronic renal failure; ASA, American Society of Anesthesiologists physical status classification; CRP, C-reactive protein; PUL.TBC, pulmonary tuberculosis−1, preoperative; 0, on the day of surgery; 1.1, on the first day after surgery; 3, on the 3rd day after surgery*.

**Figure 1:**
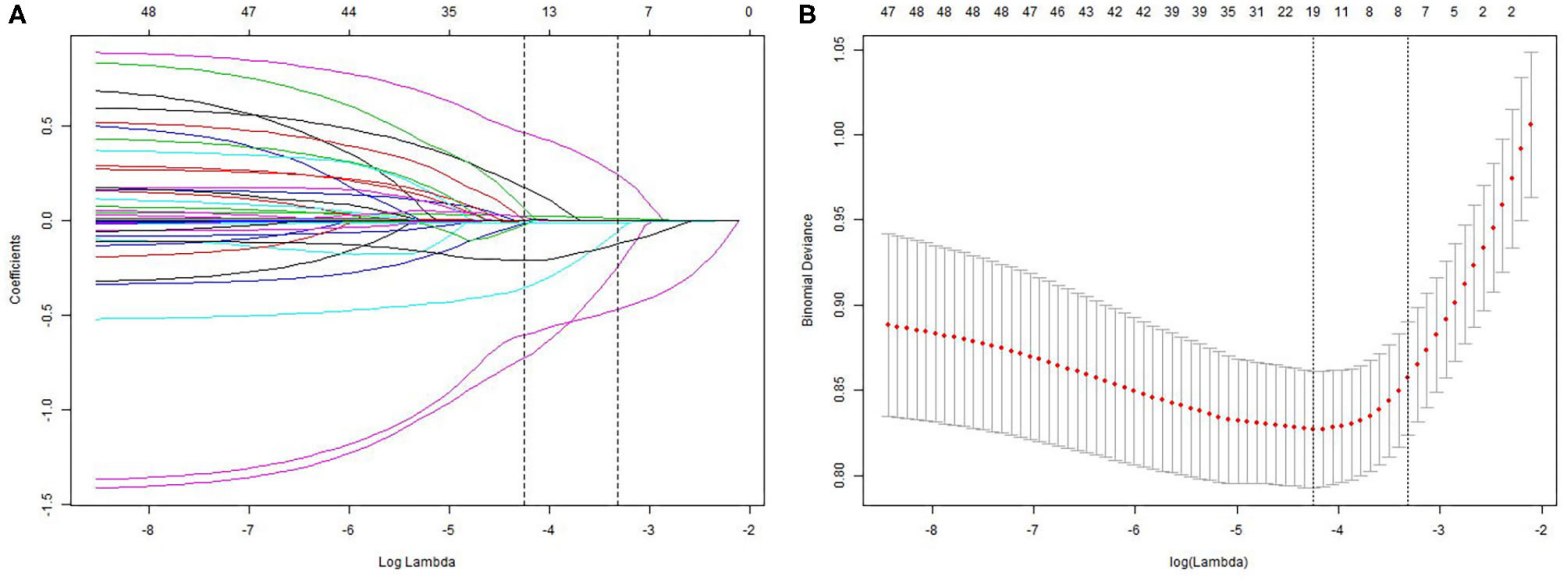
Least absolute shrinkage and selection operator (LASSO) analysis: **(A)** – Optimal parameter (lambda) selection in the LASSO model; **(B)** – demographic and clinical feature selection using the LASSO binary logistic regression model.

We analyzed the LASSO logistic regression model in the glmnet R package and determined the optimal λ value by cross-validation, with the number of folds set to 10, as shown in Figures 1A,B. The ordinate represents the target parameter, the lower abscissa represents the log(λ), and the upper abscissa represents the number of non-zero coefficients in the model at this time point. Since it is cross-validation, for each λ value, a CI for the target parameter can be obtained around the mean value of the target parameter shown as the red dot. The two dotted lines in Figure 1 represent two special λ values, i.e., lambda.min and lambda.1se. lambda.min is the λ value of the mean value of the smallest target parameter among the λ values. lambda.1se is the λ value of the most compact model obtained within a variance range of lambda.min. When lambda.1se is selected as 0.0363, the variables that enter the model are AGE, ASA, DIAGNOSIS, PLATELET.3, CHOLESTEROL.3, ALBUMIN.1, and ALBUMIN.0. As the λ value changes, the model variables are screened, as shown in Figure 1B. Each curve in Figure 1B represents the trajectory of each independent variable coefficient, with the ordinate as the value of the coefficient, the lower abscissa the log (λ), and the upper abscissa the number of non-zero coefficients in the model at this time point. As the value of λ increases, the degree of compression of the model increases, the number of independent variables entering the model decreases, and the function of the model to select the main variable strengthens.

**Figure 2:**
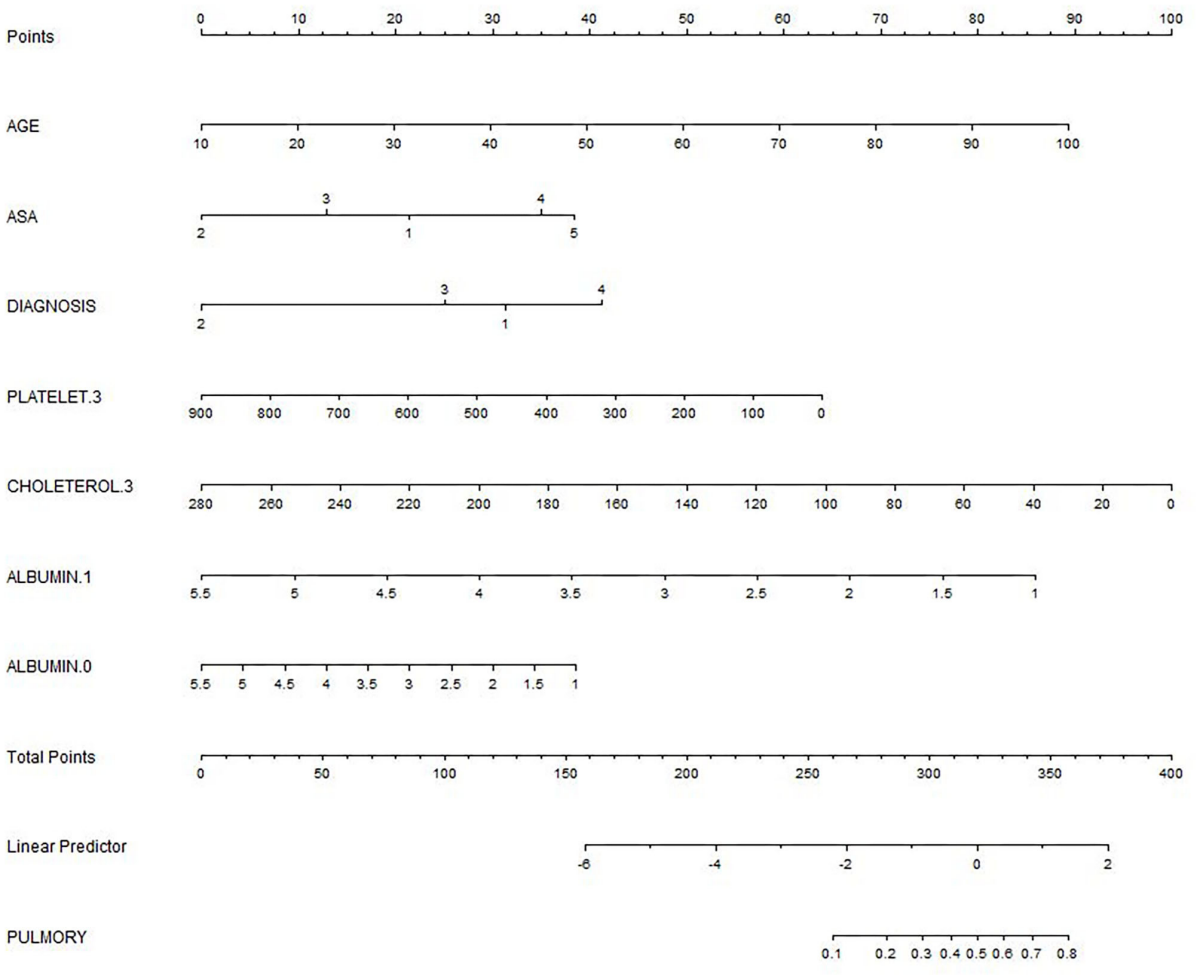
The nomogram for predicting postoperative pulmonary complications (PPCs). DIAGNOSIS: 1 Perforation; 2 Strangulation; 3 Anastomotic leakage; 4 Acute mesenteric ischemia; 0: on the day of surgery; 1.1: on the first day after surgery; 3: on the 3rd day after surgery.

We based nomogram establishment and evaluation on the independent prognostic factors shown in the logistic regression analysis, and we used R to establish a nomogram prognostic model for PPCs in patients undergoing emergency gastrointestinal surgery (Figure 2). This incorporated the variables of AGE, ASA, DIAGNOSIS, PLATELET.3, CHOLESTEROL.3, ALBUMIN.1, and ALBUMIN.0. The specific formula is:

Lambda.1se (log) [largest value of lambda such that the error is within 1 SE of the minimum]: 0.0363 (−3.3156).

Formula for calculating the score (not including the intercept): 0.01263 × AGE – 0.05786 × ASA: 2 + 0.24632 × ASA: 4 – 0.24182 × DIAGNOSIS: 2 – 0.0012 × PLATELET.3 – 0.00896 × CHOLETEROL.3 – 0.47022 × ALBUMIN.1 – 0.12434 × ALBUMIN.0.

**Figure 3:**
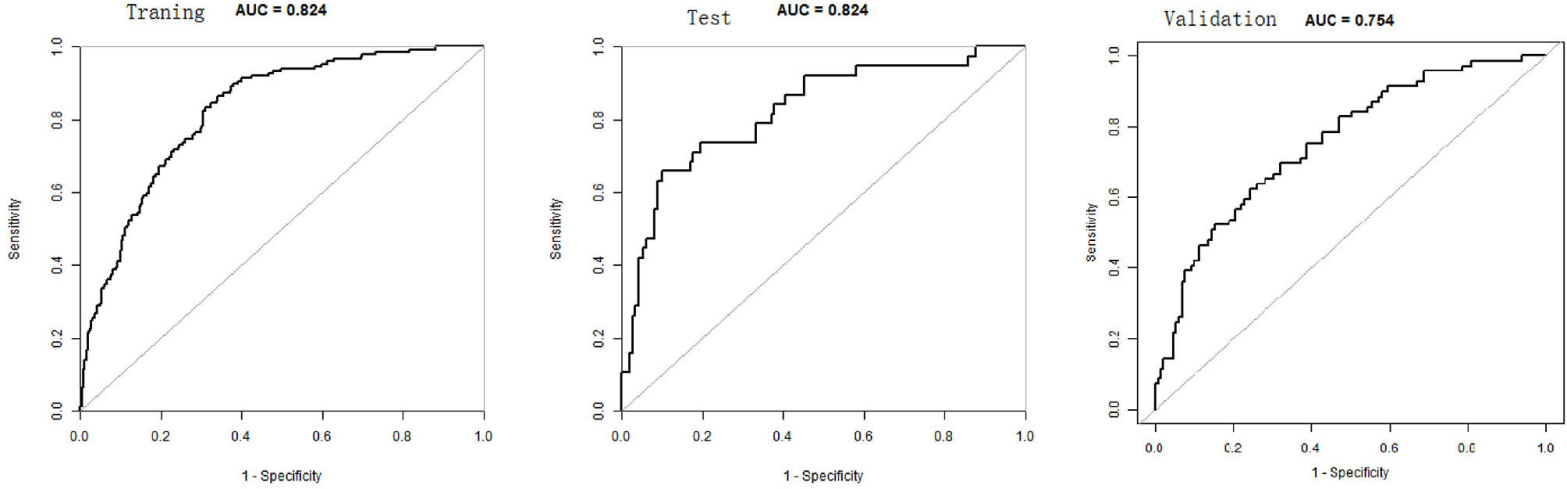
The receiver operating characteristic curve (ROC) for each nomogram.

The total score for the corresponding prediction results could be calculated by adding the scores obtained by projecting the points corresponding to each variable to the “Points” axis. In other words, we calculated the total score based on the score corresponding to each parameter on the nomogram, for predicting each patient's probability of infection. In the nomogram, we found the score corresponding to the vertical line on the “score” ruler *via* all the patients' variable values. We collected the scores of all the variable values and then found the points corresponding to the vertical line of the “predictive ruler” in the cumulative “total score” ruler. According to the score on the predictive ruler, we obtained the corresponding probability from the “incidence of PPCs” ruler, which was the patients' probability of PPCs. The area under the curve (AUC) value of the nomogram model in the training group was 0.8240, the accuracy was 0.7000, and the sensitivity was 0.8658, demonstrating that the nomogram had a higher predictive value. Also in the test group, the AUC value of the nomogram model by the variables of AGE, ASA, DIAGNOSIS, PLATELET.3, CHOLESTEROL.3, ALBUMIN.1, and ALBUMIN.0 was 0.8240, the accuracy was 0.8495, and the sensitivity was 0.8986. We also validated the results of these studies on the test dataset and the validation group dataset (Shown in Figure 3, Table 2).

**Table 2:** Nomogram model for evaluating the training, the test, and the validation groups.

	**Traning**	**Test**	**Validation**
ROC area (AUC)	0.8240	0.8240	0.7535
95%CI low	0.7896	0.7452	0.6837
95%CI upp	0.8585	0.9027	0.8233
Best threshold	−1.7099	−0.5823	−0.4386
Specificity	0.6582	0.8986	0.7557
Sensitivity	0.8658	0.6579	0.6232
Accuracy	0.7000	0.8495	0.7100
Positive-LR	2.5330	6.4912	2.5512
Negative-LR	0.2039	0.3807	0.4986
Diagnose-OR	12.4210	17.0513	5.1166
N-for-diagnose	1.9085	1.7968	2.6391
Postive-pv	0.3897	0.6250	0.5733
Negative-pv	0.9511	0.9110	0.7920

*Positive PV, positive predictive value; Negative PV, negative predictive value; Accuracy, positive - LR, positive likelihood ratio; Negative - LR, negative likelihood ratio; Diagnostic OR, diagnostic ratio; Number for Diagnostics, number of tests required for diagnosis*.

**Figure 4:**
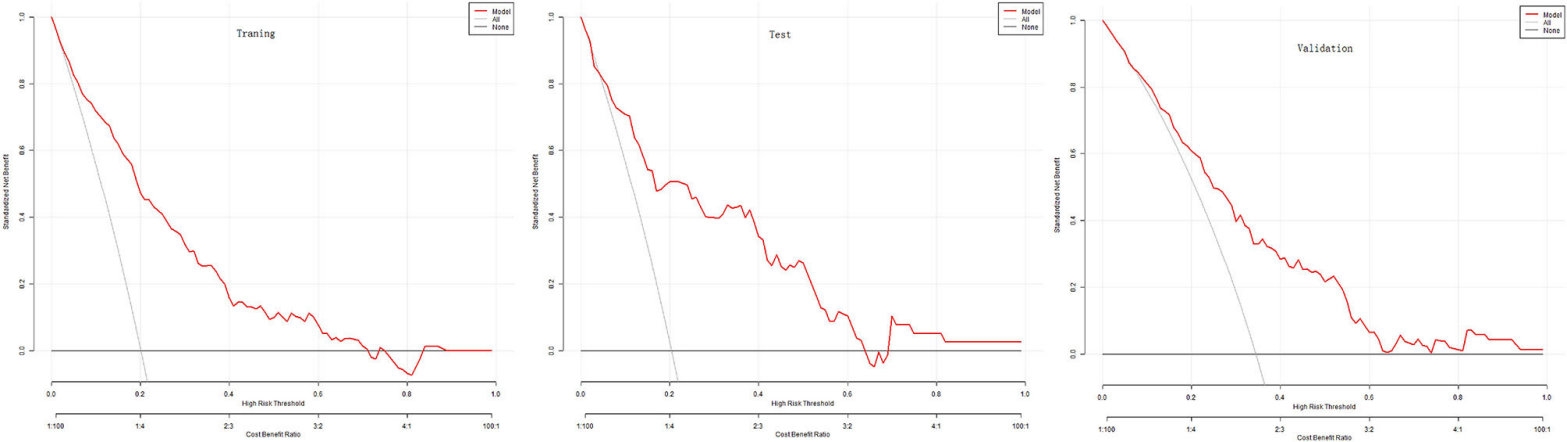
Decision curves for each nomogram.

The DCA evaluated the nomogram prognostic model with higher net income for different decision thresholds. Figure 4 shows DCA analysis in the modeled population. For example, the abscissa in Figure 4 represents the threshold probability: in the risk assessment tool, the probability of patients being diagnosed with PPCs is recorded as Pi; when Pi reaches a threshold (denoted as Pt), it is defined as positive, and treatment measures are taken. At this time, there will be benefits (advantages) for patient treatment, non-patient treatment injuries, and untreated losses (disadvantages); the ordinate is the net benefit after the reduction. The results of this study show that the model has a high benefit rate for patients. We also validated the results of these studies on the test dataset and the validation group dataset.

## Discussion

Postoperative pulmonary complications s after major surgery are an important cause of death and morbidity (4). The hospitalization time for patients at high risk for PPCs is also significantly longer than that of low-risk patients (5). A recent large-scale prospective observational study has shown that in patients who had undergone non-cardiac surgeries under general anesthesia with muscle relaxants, the incidence of PPCs was 7.6%. High-risk surgeries include intrathoracic surgery and upper abdominal open surgery. Prediction and early identification of high-risk patients with PPCs assume important clinical significance by helping clinicians take proactive measures and provide timely treatment. In this study, we analyzed clinical data from patients with diffuse peritonitis undergoing emergency gastrointestinal surgery using the LASSO regression. We found that AGE, ASA, DIAGNOSIS, PLATELET.3, CHOLESTEROL.3, ALBUMIN.1, and ALBUMIN.0 were independent risk factors for PPCs in patients with diffuse peritonitis.

Quantifying individualized PPC prediction in patients with diffuse peritonitis undergoing emergency gastrointestinal surgery remains a knotty medical problem at present. The nomogram model can quantify and visualize logistic regression results to individualize predictions of the risk of adverse clinical events. This study was based on independent PPC risk factors, such as AGE, ASA, DIAGNOSIS, PLATELET.3, CHOLESTEROL.3, ALBUMIN.1, and ALBUMIN.0, in patients with diffuse peritonitis. We established a nomogram model. The results showed that the AUC value of the nomogram model for the training group was 0.8240, the accuracy was 0.7000, and the sensitivity was 0.8658. This indicates that the nomogram had a higher predictive value. Also in the test group, the AUC value was 0.8240, the accuracy was 0.8, and the specificity was 0.8986. The results of the calibration curve and the clinical decision curve evaluation models were also good.

The preoperative factors age and ASA classification were independent risk factors for PPCs in these patients, and this was consistent with the results reported in several previous studies (6–8). The results of this study indicated that ASA scores III and IV increased the weight of the nomogram model score by approximately 13 and 35, respectively.

Moreover, studies have shown that preoperative Platelet to Lymphocyte Ratio (PLR) and Neutrophil to Lymphocyte Ratio (NLR) are good prognostic factors for PPCs and overall survival (OS) in patients with Nonsmall-Cell Lung Cancer (NSCLC) undergoing radical lung cancer surgery (9). In this study, we also found that a platelet value of 200 on the third day would increase the weight of the nomogram model score by 50.

High-density lipoprotein cholesterol (HDL-C) levels appear to be one of the risk factors for postoperative nosocomial infections (10). Studies (11) have also shown that a decrease in serum cholesterol levels is a poor prognostic factor in patients with severe community-acquired pneumonia. In this study, we also found that cholesterol levels were associated with PPCs. The cholesterol value being 100 on the third day would increase the weight of the nomogram model score by approximately 62.

Moreover, studies (12) have shown that perioperative serum albumin changes are predictive factors of PPCs in patients with lung cancer. Similarly, preoperative albumin levels can be used as predictive factors of PPCs after elective laparoscopic gastrectomy (13). In our study, we also found that albumin levels were associated with PPCs. An albumin value of 1.5 on the first day would increase the weight of the nomogram model score by approximately 78.

Although we have included several prognostic factors to establish a more accurate prediction of postoperative survival in patients with non-metastatic bowel cancer, there remain several limitations to this study. First, there was only one source for the sample. Since there was only one disease involved in this study, the model for the overall population needs further improvement. Second, in this study, we use internal verification. Therefore, the findings are not necessarily applicable to external data. Third, this study is retrospective, so there may have been selection bias in the data collection. Finally, PPC diagnosis is dependent on clinical judgment and confirmed by imaging and laboratory examination. Therefore, some lung infections, pleural effusion, and atelectasis that do not show obvious symptoms may be overlooked, leading to an underestimation of PPC incidence.

## Conclusion

Our research results showed that AGE, ASA, platelets, cholesterol, and ALBUMIN are high-risk factors for pulmonary complications after gastrointestinal emergency surgery in patients with peritonitis. Moreover, the nomogram established by the above factors can quantitatively evaluate the likelihood of pulmonary complications.

## Data Availability Statement

Publicly available datasets were analyzed in this study. This data can be found here: Data is available at BioStudies database (https://www.ebi.ac.uk/biostudies/studies/S-EPMC6034864), accession numbers: S-EPMC6034864.

## Ethics Statement

The studies involving human participants were reviewed and approved by the Ethics Committee at the First Affiliated Hospital of Zhengzhou University (2021-KY-677). Because it was a retrospective study, the Ethics Committee exempted patient informed consent. Written informed consent for participation was not required for this study in accordance with the national legislation and the institutional requirements.

## Author Contributions

All authors contributed to the article, data analysis, drafting or revising of the article, final approval of the version for publication, agreed to be accountable for all aspects of the work, prepared Figures 1–4, and approved the submitted version.

## Conflict of Interest

The authors declare that the research was conducted in the absence of any commercial or financial relationships that could be construed as a potential conflict of interest.

## Publisher's Note

All claims expressed in this article are solely those of the authors and do not necessarily represent those of their affiliated organizations, or those of the publisher, the editors and the reviewers. Any product that may be evaluated in this article, or claim that may be made by its manufacturer, is not guaranteed or endorsed by the publisher.

## Abbreviations

Hb: hemoglobin
HTN: hypertension
DM: diabetes mellitus
CRF: chronic renal failure
ASA: American Society of Anesthesiologists physical status classification
CRP: C-reactive protein
PUL.TBC: pulmonary tuberculosis
−1: preoperative
0: on the day of surgery
1.1: on the first day after surgery
3: on the 3rd day after surgery.
